# Cross-Sectional Study: Assessing the Presence of Stx2e-Producing *E. coli* Virotypes in Samples of Oral Fluid of Growers and Fatteners

**DOI:** 10.3390/pathogens14030261

**Published:** 2025-03-06

**Authors:** Ana Trbovc, Matevž Pušnik, Tim Šteferl, Melita Hajdinjak, Marina Štukelj

**Affiliations:** 1 Clinic for Reproduction and Large Animals, Clinic for Ruminants and Pigs, Veterinary Faculty, University of Ljubljana, Cesta v Mestni log 47, 1000 Ljubljana, Slovenia; at8963@student.uni-lj.si (A.T.);; 2 Laboratory of Applied Mathematics and Statistics, Faculty of Electrical Engineering, University of Ljubljana, Tržaška cesta 25, 1000 Ljubljana, Slovenia; melita.hajdinjak@fe.uni-lj.si

**Keywords:** edema disease, oral fluid, *E. coli*, prevalence, biosecurity

## Abstract

Edema disease is a multifactorial infectious disease caused by specific *E. coli* virotypes possessing fimbriae F18 and toxin Stx2e that cause significant losses in the post-weaning period. The aim of this study was to assess the presence of Stx2e-producing *E. coli* verotypes in Slovenian commercial pig farms in relation to the biosecurity and technological measures undertaken by the owners. Samples of oral fluid were collected from growers and fatteners at 5–6 weeks, 7–8 weeks, 12 weeks and 14 weeks of age on 37 commercial pig farms, using the Verocheck^^®^^ diagnostic kit for the real-time PCR detection of Stx2e. The results of RT-PCR and the questionnaire were statistically analyzed. The prevalence of *E. coli* strains producing Stx2e was 64.9%. Statistically significant association between the prevalence of Stx2e producing *E. coli* strains and the type of the farm and feed origin was proved. No association was found between prevalence and farm size, presence of quarantine or previous outbreaks of edema disease. None of the studied age groups showed a statistically significant dominance in prevalence compared to other age groups, which contradicts the current theoretical data. Further studies are needed to estimate the proportion of Stx2e produced by the EDEC pathotype compared to other *E. coli* strains.

## 1. Introduction

Edema disease is a multifactorial infectious disease caused by *E. coli* virotypes with the ability to produce Stx2e toxin. The most common causative serotype isedema disease *E. coli* (EDEC), a subgroup within the pathotype called Shiga toxin-producing *E. coli* (STEC), which possesses fimbrial adhesin F18 [1] or fimbrial adhesin AIDA [2] and produces both Shiga toxin 2e (Stx2e), verotoxin 2e (VT2e), EAST1 (enteroaggregative *Escherichia coli* heat-stable enterotoxin 1) and alpha-hemolysin [3,4,5]. Due to interbacterial exchange of genetic material encoding virulence factors, edema disease can also be caused by other *E. coli* serotypes [6,7], as discovered in South Africa, where the *E. coli* O149:K91:K88 (F4) strain was shown to be associated with clinical edema disease [8].

The majority of clinical cases of edema disease is observed after weaning, when the pigs are 4–12 weeks old [9]. Younger categories of pigs do not develop edema disease due to adequate maternal antibody levels [3] and the absence of complementary receptors for bacterial binding factors in the small intestine [10,11]. Although it is possible for pigs to become infected by a contaminated environment, water, feed or transport vehicles or interaction with other animal species [2], the most common cause is ingestion [3,4]. Diets exceptionally rich in carbohydrates and protein, especially when combined with ad libitum feeding, cause an imbalance in the physiological gut microflora and lead to an increased population of pathogenic bacterial strains in the pigs’ small intestines, indirectly influencing the prevalence of edema disease. In some cases, a high-protein diet with very little nutritional supplementation may also predispose sows to edema disease [6]. Another known indirect factor that increases the prevalence of edema disease is concurrent infection with rotaviruses [12].

Once ingested, the causative *E. coli* serotypes proliferate in the pig’s small intestine. The subsequent colonization is enabled by the interaction between specific adhesin-binding receptors located on host enterocytes at the base of the villi and subunit B of the F18 adhesive fimbriae that are located on the bacterial surface [3,6,11,13]. Pigs without receptors for the F18 fimbrial adhesive protein are immune to disease [6,13,14], while pigs with receptors show individual differences in the amount of toxin absorbed, suggesting that the toxin may cross the intestinal barrier via a non-specific mechanism [11,15]. Clinical signs are caused by Stx2e, which is absorbed through damaged intestinal mucosa into the systemic circulation and smooth muscle cells of the arteriolar tunica media, where it interferes with ribosomal RNA function, preventing the synthesis of new proteins and ultimately leading to cell apoptosis [3,6,11,16]. This results in endothelial swelling and vacuolization of host blood vessels; subendothelial deposition of fibrin; perivascular edema; microthrombus formation; tunica media necrosis; endothelial proliferation; and, in some cases, damage to the submucosal nerve plexus [3,6].

The most commonly observed clinical signs include edema of the eyelids and forehead, blindness, partial ataxia, incoordination, staggering gait, respiratory distress, rowing movements in lateral recumbency, stupor and high mortality, especially in pigs in the best body condition. In subclinically affected pigs, only growth retardation can be observed compared with healthy counterparts [3,5,17]. Pigs recovering from acute edema disease may develop a condition formerly known as cerebrospinal angiopathy, which is associated with nervous clinical signs such as circling, head twisting and limb muscle atrophy with progressive weakness. Atypical edema disease with terminal diarrhea and fatal shock are less common courses of edema disease [3].

Diagnosis of edema disease includes pathomorphological examination of deceased pigs, as well as laboratory tests. Post-mortem findings are evident, even in subclinically affected pigs [6], and include pathognomonic edema of the greater curvature of the stomach between the mucosa and the muscular layer and gelatinous edema of the cardia or fundus up to 2 cm thick [3,18]. Other common findings include edema of the mesocolon [3,6,18], edema of the mesentery of the small intestine, edema of the gallbladder [3,18], edema of the throat and renal capsules [3,6,18] and edema of the lungs [3]. Relevant samples for laboratory diagnostics include feces, rectal swabs or oral fluid from pigs, all ideally collected during the acute phase of the disease, while a mucosal swab from the distal jejunum, ileum or colon is collected from deceased pigs [19]. In particular, feces are considered the most appropriate sample type [5,19]. Microbiological detection methods are gradually being replaced by molecular methods involving a combination of bacterial DNA isolation and a duplex PCR assay to determine the presence and amount of bacteria. In the case of porcine edema disease, the genes for fimbrial adhesion protein F18 and verocytotoxin Stx2e are detected simultaneously [5,19]. One of the most commonly used novel methods is a fluorescent PCR assay, which uses the 5’ activity of Taq polymerase. 

Edema disease is economically important, with a worldwide incidence of 10–30% [6] and a mortality rate of 50–90% in affected pigs [3,11]. Treatment of edema disease is rarely successful, so preventive measurements of good husbandry and biosecurity, such as all–in–all out method, appropriate environmental temperature and humidity, proper hygiene, prevention of biofilm formation, limited access to feed, correct nutrient ratios in feed and preventive vaccination of piglets or growers against edema disease, are very important [3].

To date, no studies have been conducted on the prevalence of edema disease in Slovenia, and very few studies have been published on the prevalence of edema disease abroad. Tseng et al. reported a 65.3 % prevalence of the STEC pathotype in American fatteners in a longitudinal study published in 2012, and Berger et al. reported a 37.4 % prevalence of the EDEC pathotype in German growers and 53.5 % samples positive for the Stx2e toxin [5,20]. Baek et al. mentioned that out of 880 *E. coli*-associated pig infections between 2012 and 2014, there were 52 confirmed and 2 suspected cases of porcine edema disease in the Republic of Korea [21]. The aim of this cross-sectional study was to assess the current proportion of farms affected by edema disease, including growers and fatteners, in Slovenian commercial pig farms and to evaluate the influence of specific technological measures and biosecurity on the proportion of farms affected by edema disease, including the size and type of farm, the presence of quarantine measures, the origin of feed and the presence of previous outbreaks of edema disease. The working hypothesis included the distribution of the pathogen among growers and fatteners based on their age. The study used single oral fluid sampling as a non-invasive sampling method for the detection of Stx2e-encoding *E. coli* strains. 

## 2. Materials and Methods

### 2.1. Study Population

**Figure 1:**
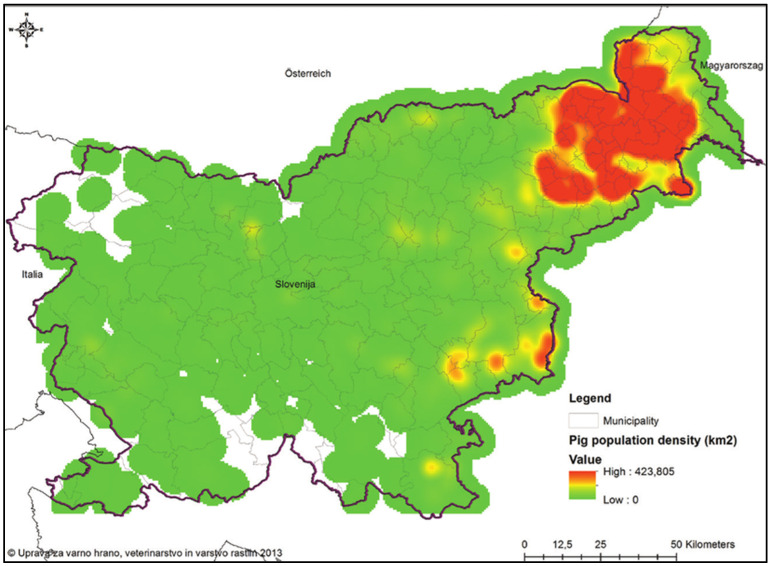
Domestic pig population density in Slovenia in 2013 [22].

Between January and May 2024, 37 commercial pig farms from the Štajerska, Prekmurje and Dolenjska regions were selected and purposively sampled for this study. According to the data from the Statistical Office of the Republic of Slovenia, the total number of pigs kept in Slovenia in 2023 was approximately 196,000, of which 33,000 were pigs with a body weight of 20–50 kg, which corresponds to the target population for this epidemiological study. The majority of the pig population in Slovenia consists of family farms with fewer than 10 pigs in total, while larger commercial farms are located mainly in the northeastern part of Slovenia in the Prekmurje and Štajerska regions (Figure 1). Due to the small overall pig population in Slovenia and the uneven distribution of larger farms between regions, all participating farms were selected on the basis of the inclusion criteria for non-probability sampling after prior consultation with local veterinarians. We estimated the ideal sample size to be 73 samples using Cochran’s sample size formula (confidence interval, 95%; margin of error, 5%; z-score, 1.96; population proportion, 5%); during the sampling process, a total of 170 pens were sampled for oral fluid.

### 2.2. Study Design

All farms selected for this study had to meet the requirement of having at least 20 breeding sows to ensure sufficient samples of all desired age groups or they were excluded from the research. Four farms within the same region were sampled per day, each of them by a separate sampler or by the farmers themselves to ensure proper external biosecurity. All farmers were contacted before sampling and interviewed by telephone or email or on site regarding general husbandry information, biosecurity measures, technology, general herd health and health status regarding edema disease. For statistical analysis, 36 fully completed surveys were used. To assess the initial hypothesis, responses on the type of farming (indoor-only farms versus farms where pigs also have access to outdoor pens), farm size (farms with more than 500 pigs versus farms with fewer than 500 pigs), the presence of quarantine measures (farms that have quarantine measures versus farms that do not), origin of feed (farms that use commercial feed versus farms that mix their own feed) and previous outbreaks of edema disease (farms that have never had an outbreak of edema disease versus farms that have had outbreaks of edema disease in the past) were used. The target population was divided into 4 age groups (5–6 weeks, 7–8 weeks, 12 weeks and 14 weeks). Each age group was sampled as a separate epidemiological unit within the farm in order to compare the prevalence between different age groups of growers and fatteners. If certain age groups listed above were not available for sampling, several pens of the remaining age groups were sampled, and the results were defined as positive for the farm if at least one of the sampled pens for the age groups tested positive for the Stx2e toxin. A sufficient volume of oral fluid for the detection of Stx2e toxin was required by the FTA protocol leaflet, which proved to be problematic in 5–6-week-old pigs, despite adequate exposure time. Only epidemiological units in which specimens produced a sufficient amount of sample were used in the research. If a sample was contaminated during the sampling process or compromised in any other way, it was excluded from the research before inoculation and detection of Stx2e toxin.

### 2.3. Sampling and Diagnostic Method

Verocheck^®^ diagnostic kits were used for non-invasive sampling of oral fluid in growers and fatteners. Each kit consisted of 4 cotton ropes, 4 plastic bags, 4 Falcon tubes, 4 plastic pipette tubes of 100 µl, 4 pairs of protective gloves, 4 packs of FTA (Fast Technology for Analysis of Nucleic Acids) cards, an FTA protocol leaflet and a HIPRA envelope. In each designed age group of pigs, one pen was randomly selected for sampling. The rope was hung within the reach of the pigs but high enough to avoid potential environmental contamination. The pigs were allowed to interact with the ropes for a minimum of 30 minutes to ensure an adequate amount of oral fluid sample; then, the ropes were removed and transferred to plastic bags, within which the oral fluid was manually extracted from the ropes and transferred to plastic Falcon tubes. The latter were shaken 3 times before 100 µL of the sample was inoculated onto FTA cards using pipette tubes. The sample had to be inoculated within the circle drawn on the FTA cards and allowed to dry for at least one hour at room temperature before the flap was folded down. Each FTA card was recorded with the name of the farm and the age of the sampled pigs before being placed in the appropriate plastic bag with the desiccant and placed in the HIPRA envelope with the leaflet containing detailed information about the farm. Separate gloves, pipettes and FTA cards were used when changing samples to avoid cross-contamination of samples. The envelopes were sent to the HIPRA diagnostic laboratory at Avinguda de la Selva 135, 17170 Amer in Girona, Spain, where a modified real-time PCR assay was performed.

TaqMan Universal Master Mix 2x from Applied Biosystems was used in a modified fluorescent real-time PCR assay to determine the quantity and quality of bacterial nucleic acid in samples. The kit contains AmpliTaq Gold DNA polymerase, AmpErase uracil-N-glycosylase (UNG), dNTPs with dUTP, 6-carboxy-X-rhodamine (ROX) as an internal passive fluorogenic reference and optimized buffer components. The 5’ nuclease assay was performed in 20 µl volumes containing 600 nM of each factor primer (one factor per test tube), 75 nM of each 16S rRNA primer, 200 nM of each probe labeled with FAM and TET reporter dyes (factor and 16S rRNA probe, respectively), 10 µL of TaqMan Universal Master Mix and sterile distilled water added to a total volume of 18 µL. These reagent mixtures were added to PCR tubes in batches of 80–100 tubes per factor and stored at −20 °C. Before starting the assay, 2 µL of diluted template lysate was added. Tubes were sealed with Applied Biosystems MicroAmp Optical Caps. Batch control was performed for each run, as the assay included both non-template controls and positive controls for each factor. Thermal cycling consisted of initial steps at 50 °C for 2 min, as required for optimal AmpErase UNG enzyme activity, and 95 °C for 10 min, as required to activate the AmpliTaq Gold DNA polymerase and deactivate the AmpErase UNG enzyme. This was followed by 40 cycles of 95 °C for 15 s and 60 °C for 1 min. Amplification was performed on an Applied Biosystems ABI prism 7700 Sequence Detection System. The last 3 readings of the fluorescence during the extension step of each PCR cycle were used to calculate the ΔRn value. The threshold cycle (Ct) was calculated by software [23]. Samples with a Ct value below 38.5 were defined as positive for the Stx2e toxin and further divided into 4 groups according to the amount of pathogenic genetic *E. coli* material detected as NEG (−), POS (+), POS (++) and POS (+++).

### 2.4. Statistical Analysis

All the statistical analyses were performed using R, version 2023.09.1 by the R Foundation for Statistical Computing [24]. 

Data from 36 farms that completed the survey and provided PCR test results were statistically analyzed. Every farm where at least one of the sampled categories of pigs was proven positive for edema disease caused by *E. coli* serotypes was defined as positive for edema disease. As the data were binary (positive for edema disease: yes/no), only statistical tests with count data could be used to compare the proportions of different groups of animals or farms. Fisher’s exact test, the test for equal or given proportions (also known as the chi-squared test) and the Mann–Whitney test for count data were used to test the null hypothesis that the proportion of edema was equal in the two samples being compared [25]. It is known that Fisher’s exact test is usually the best choice when one of the groups being compared is quite small (fewer than 10), while the other may be large (well above 10). The equal proportions test is more appropriate when both groups have much larger samples, and the Mann–Whitney test applies to ordinal or continuous data and may not be the perfect choice for a simple yes/no result. We used 0.05 as the significance level.

A statistical power of 0.8 at a significance level of 0.05 for Fisher’s exact test was estimated by Monte Carlo simulations to require a sample size of about 25–28 per group to detect an increase in proportion from 20% to 50%. In contrast, the test for equal or given proportions requires about 37 pigs per group (according to the large sample formula using power.prop.test in Base R), and the Mann–Whitney test requires an even larger sample size of about 64 pigs for the same power (using pwr.t.test in Base R). Consequently, the results of this study are based primarily on the results of Fisher’s exact test.

Given the binary nature of the outcome of interest (edema disease), logistic regression analyses [26] were conducted as a supporting method to determine whether there were statistically significant differences in the log odds of edema disease based on farm size, type of husbandry, type of feed, quarantine practices and past outbreaks of edema disease. Pig age was included as a confounder in the model due to its potential association with both the risk indicators and disease proportion. Odds ratios (ORs) for the observed risk indicators, along with their corresponding 95% confidence intervals (CIs), were calculated to assess the strength and precision of these associations.

## 3. Results

### 3.1. Survey

Interviews were conducted with 36 farmers: 25 from the Štajerska region, 9 from the Prekmurje region and 2 from the Dolenjska region. A total of 32 surveys were completed in full, and 4 were completed in part, although still with sufficient information for statistical analysis. The survey of farm number 22 was never received. Therefore, a total of 36 surveys were used for the statistical analysis. The results of the surveys are presented in Table 1.

### 3.2. RT-PCR

A total of 37 RT-PCR results were received from Girona. On 24 farms there was at least one positive sample per category of pigs, indicating a 64.9% prevalence of edema disease in Slovenia. The results of the RT-PCR assay as received from Girona are presented in Table 2.

On farms numbered 3, 8, 15 and 29, despite the adequate sampling time, not enough samples were obtained from the youngest age group of pigs, which prevented successful FTA inoculation. On farm number 14, the 12-week-old pigs bit off the rope and dragged it to the ground, where it was contaminated and, therefore, useless for the RT-PCR assay. On farms numbered 3, 4, 5, 8, 10, 12–15, 20, 21, 24–29 and 33–36, some age groups of pigs were missing, so only the age groups present during the research were sampled and used in the RT-PCR assay.

### 3.3. Statistical Analysis

#### 3.3.1. The Size of the Farm

**Figure 2:**
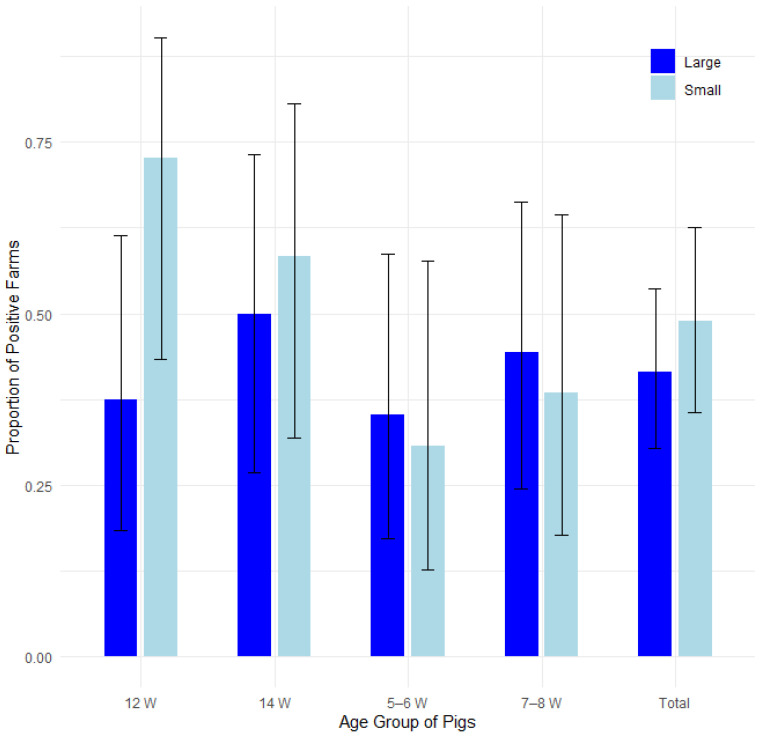
Proportion of positive farms by farm size and pig age group with 95% confidence intervals.

**Figure 3:**
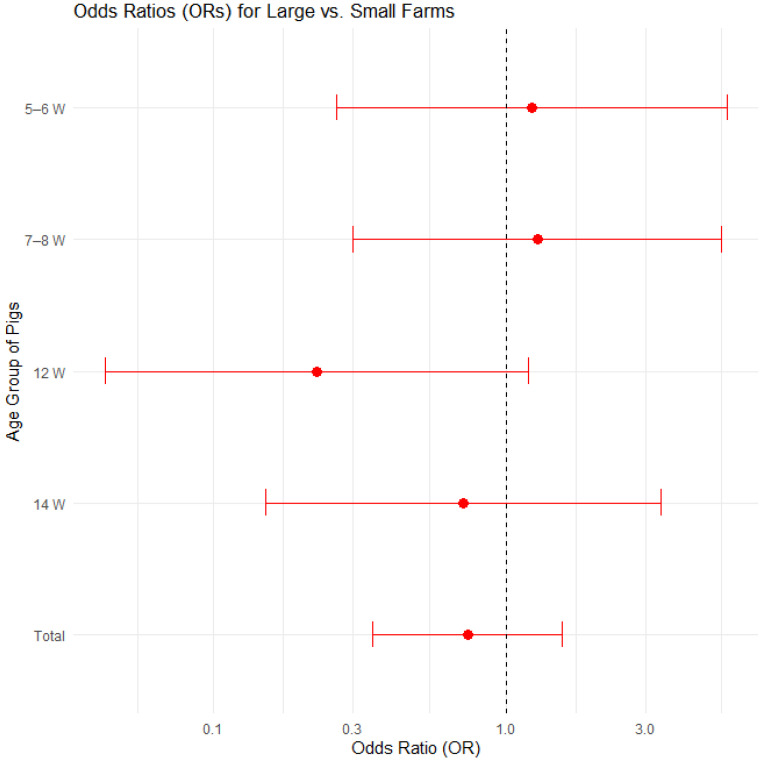
Odds ratios (ORs) with 95% confidence intervals for edema disease presence on large farms vs. small farms.

The farms were divided into two groups based on the total number of pigs from all categories: large farms (farms with a total of at least 500 pigs) and small farms (farms with a total of fewer than 500 pigs). The proportion of farms affected by edema disease was compared among 20 large farms and 16 small farms. Figure 2 displays the proportions of positive cases by farm size and pig age group, with 95% confidence intervals in a bar plot, while Figure 3 presents the corresponding odds ratios with their 95% confidence intervals.

No statistically significant difference was found between large farms and small farms using Fisher’s exact test for count data, a test of equal or given proportions and Mann–Whitney test. The farms were statistically evaluated both as a whole and for specific age groups of sampled pigs, as shown in Table 3.

A logistic regression model (binomial family) was used to additionally assess the effects of farm size (small vs. large) and pig age group on edema disease risk. The results are presented in Table S1 and Figure S1 in the Supplementary Materials. None of the predictors was statistically significant (*p* > 0.05). The odds ratio for small versus large farms was OR = 1.36 (95% CI: 0.64–2.91), indicating no clear difference in edema disease proportion by farm size. Similarly, age-group effects had wide confidence intervals overlapping 1.0, indicating high uncertainty. Thus, we found no strong evidence that farm size or age group is associated with edema disease in this dataset.

#### 3.3.2. The Type of Husbandry

**Figure 4:**
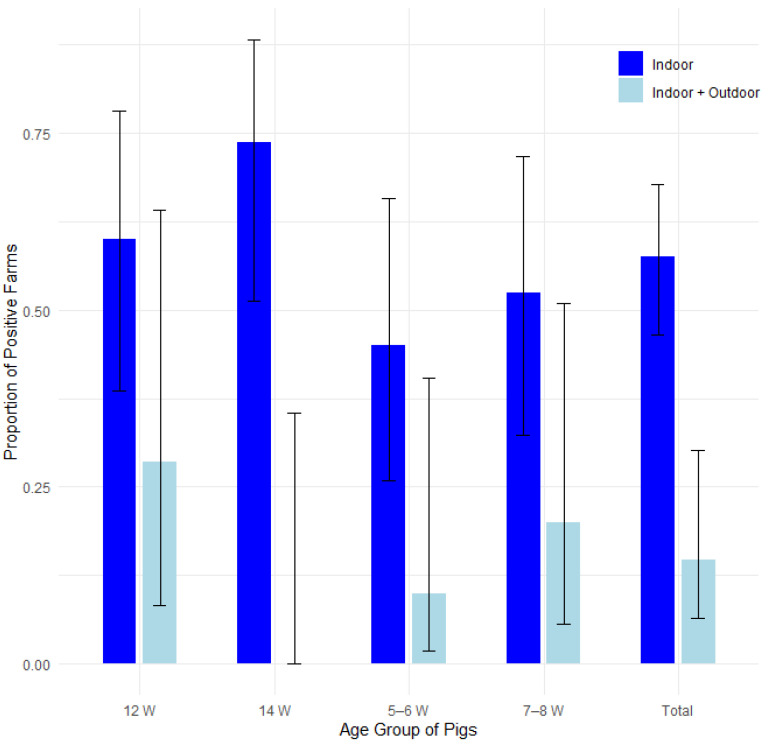
Proportion of positive farms by type of husbandry and pig age group with 95% confidence intervals.

**Figure 5:**
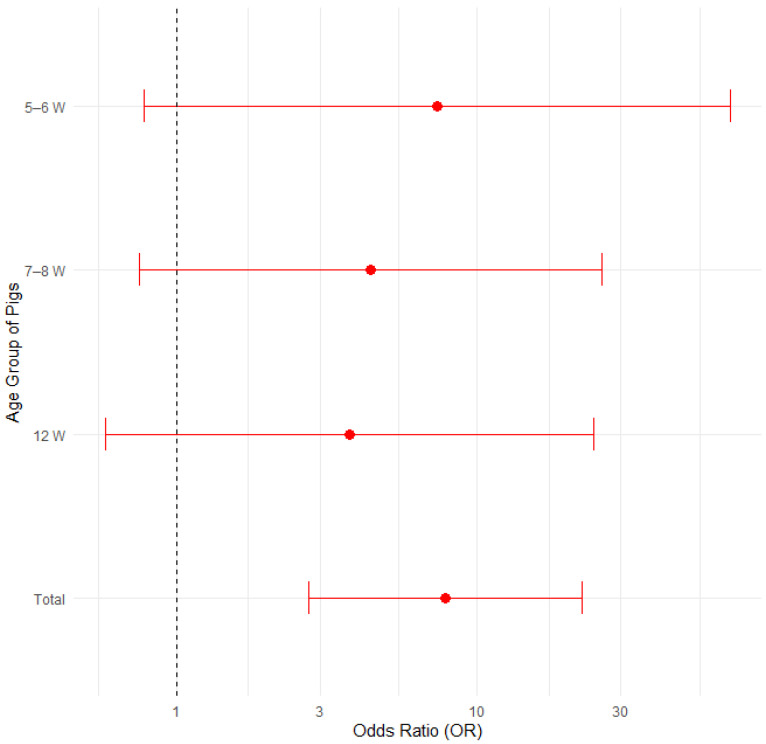
Odds ratios (ORs) with 95% confidence intervals for edema disease presence in farms without access to outside runs vs. farms with access to outside runs.

The proportion of affected farms was compared among 22 farms where pigs had no access to outside runs and 14 farms where pigs had access to outside runs (Table 4). Figure 4 displays the proportions of positive cases by type of husbandry and pig age group, with 95% confidence intervals in a bar plot, while Figure 5 presents the corresponding odds ratios with their 95% confidence intervals.

A statistically significantly higher proportion of edema disease was shown on farms where pigs had access to outside pens when compared to farms where they did not, in farms as whole (*p* < 0.01) and in the group of 14-week-old pigs (*p* < 0.05). Other age groups, when statistically evaluated, showed no such difference. Data were statistically evaluated using Fisher’s exact test, the test of equal or given proportions and the Mann–Whitney test. 

Logistic regression analysis revealed that the type of husbandry was a significant predictor of edema disease occurrence (OR = 0.13, 95% CI [0.04–0.34], *p* < 0.001). This indicates that pigs raised with outdoor access had substantially lower odds of developing edema disease compared to those in indoor-only systems. In contrast, age group did not significantly influence edema disease odds (all *p* > 0.05). The results are presented in Table S2 and Figure S2 in the Supplementary Materials. 

#### 3.3.3. Feed

**Figure 6:**
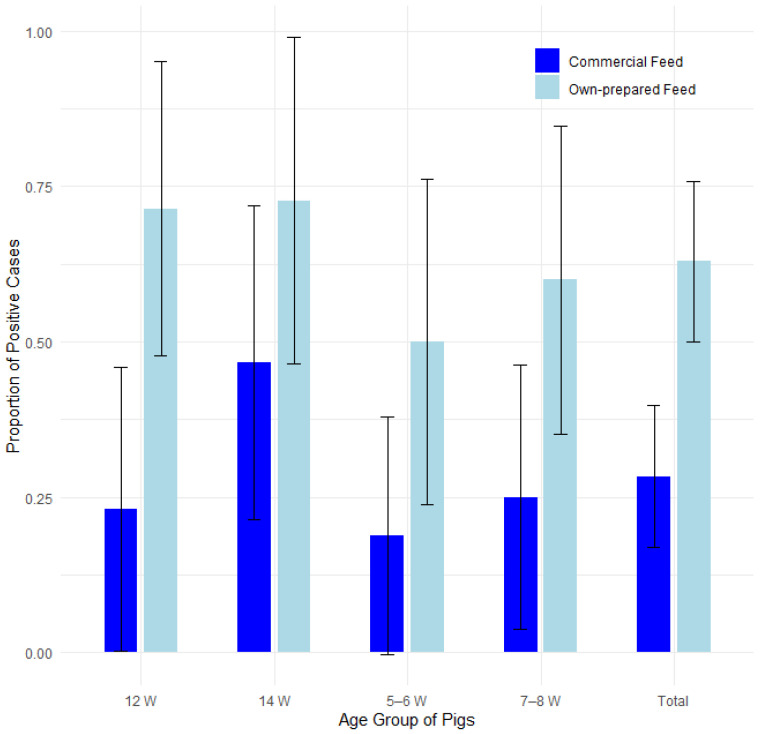
Proportion of positive farms by type of feed and pig age group with 95% confidence intervals.

**Figure 7:**
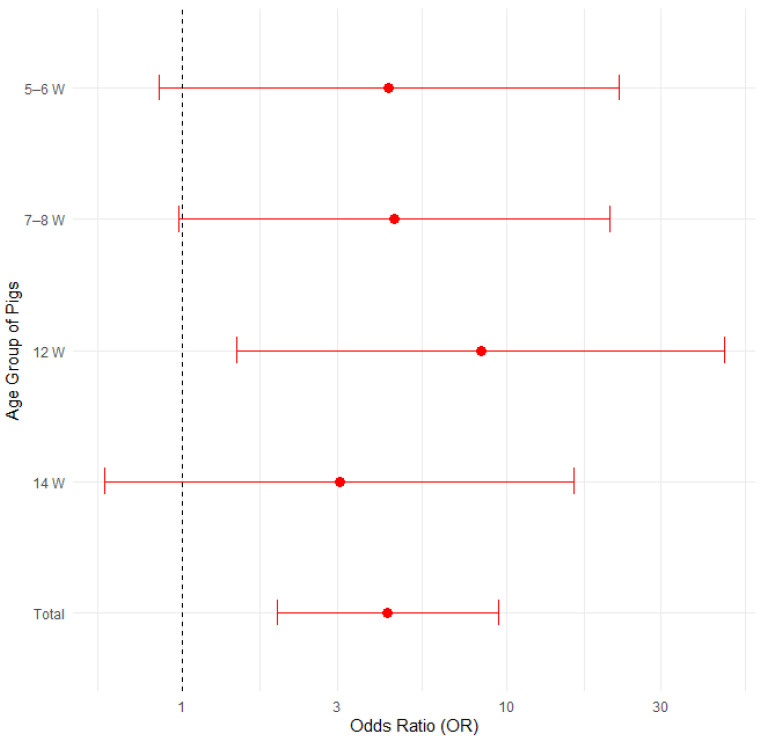
Odds ratios (ORs) with 95% confidence intervals for edema disease presence on farms where only commercial feed was used vs. farms where farmers prepared their own feed.

The proportion of farms affected was compared among 19 farms where only commercial feed was used and 17 farms where farmers prepared their own feed (Table 5). Figure 6 displays the proportions of positive cases by type of feed and pig age group, with 95% confidence intervals in a bar plot, while Figure 7 presents the corresponding odds ratios with their 95% confidence intervals.

Results of statistical analysis show that the proportion is statistically significantly higher on farms where owners prepared feed on their own compared with farms where pigs were fed commercial feed only (*p* < 0.05). The proportion of edema disease in pigs aged 12 weeks showed the same statistical trend (*p* < 0.05) when analyzed using Fisher’s exact test for count data and the Mann–Whitney test. Results were statistically analyzed using Fisher’s exact test of count data, the test of equal or given proportions and the Man–Whitney test.

Logistic regression showed that farms using self-prepared feed had 4.7 times higher odds of edema disease (95% CI: 2.12–11.02, *p* < 0.001) compared to farms using commercial feed. Additionally, pigs aged 14 weeks had significantly higher odds of edema disease compared to 5–6-week-old pigs (OR = 3.42, 95% CI: 1.08–11.58, *p* = 0.04). No significant differences were observed among the other age groups. The results are presented in Table S3 and Figure S3 in the Supplementary Materials.

#### 3.3.4. Quarantine

**Figure 8:**
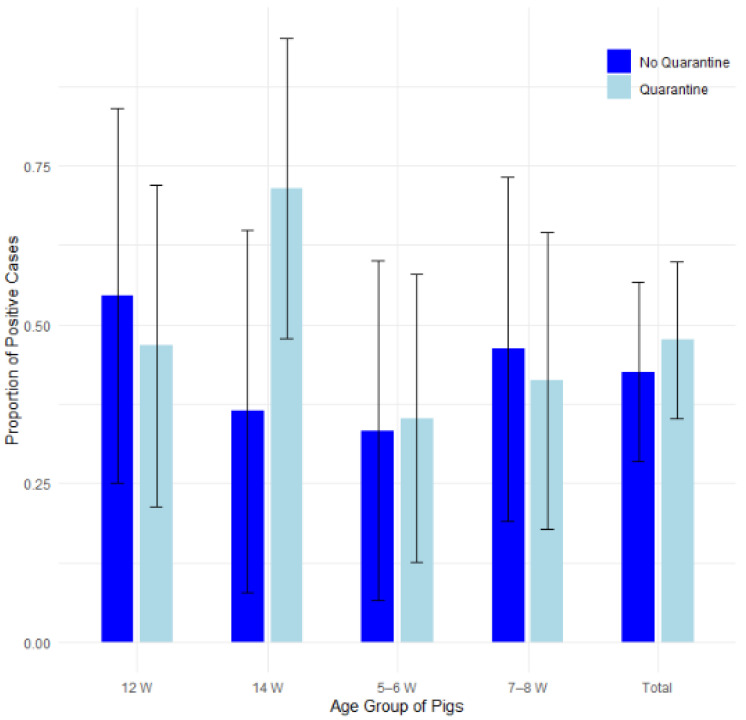
Proportion of positive farms by quarantine presence and pig age group with 95% confidence intervals.

**Figure 9:**
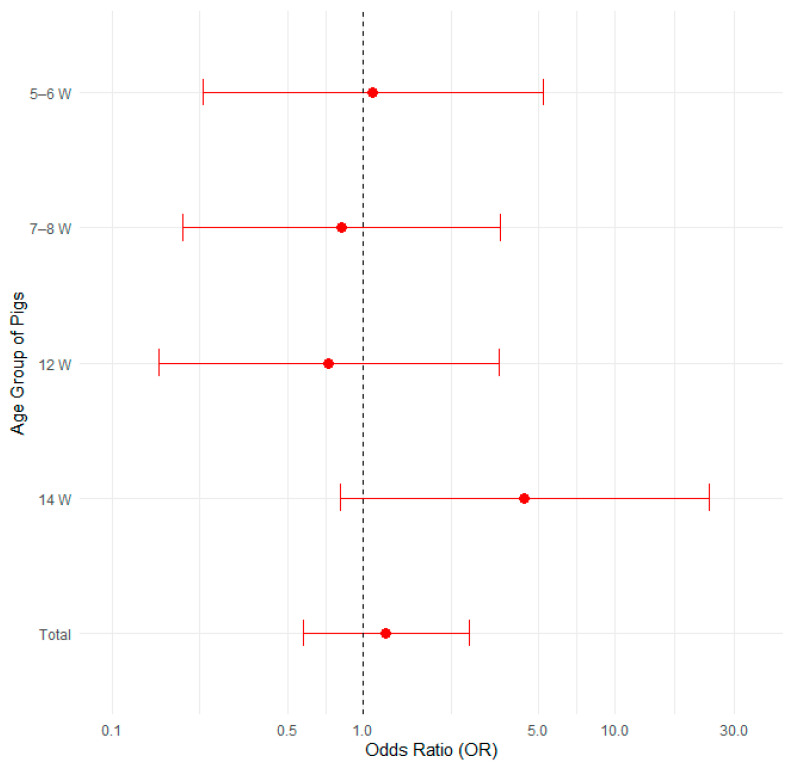
Odds Ratios (ORs) with 95% Confidence Intervals for edema disease presence in farms where biosecurity includes quarantine vs. where it does not include quarantine.

The proportion of disease presence was compared among 22 farms that implemented quarantine for newly arrived pigs and 13 farms that did not (Table 6). Farm number 16 was not included in the statistical analyses, as the farmer breeds his own gilts, therefore not introducing newly purchased pigs to his farm. Figure 8 illustrates the proportions of positive cases by quarantine presence and pig age group, with 95% confidence intervals in a bar plot, while Figure 9 presents the corresponding odds ratios with their 95% confidence intervals.

Fisher’s exact test, the test of equal or given proportions and the Mann–Whitney test revealed no statistically significant differences between the groups (Table 6).

A logistic regression analysis was conducted to evaluate the impact of quarantine and pig age group on the presence of edema disease. The findings indicated that neither the quarantine status nor the age groups (*p* > 0.05, confidence intervals overlapping 1.0) were significantly associated with edema disease presence. The results are presented in Table S4 and Figure S4 in the Supplementary Materials.

#### 3.3.5. Past Outbreaks of Edema Disease

**Figure 10:**
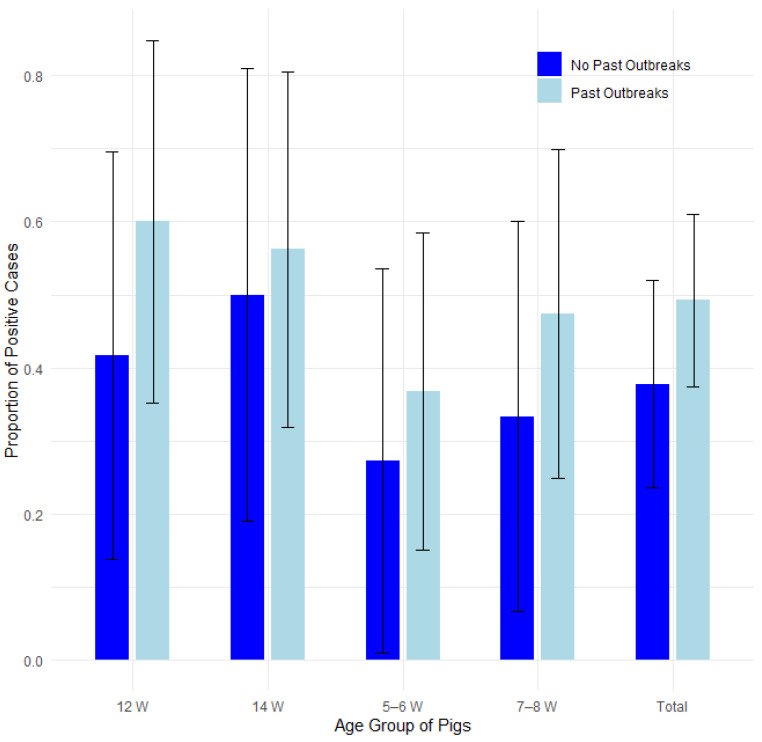
Proportion of positive farms by past outbreaks and pig age group with 95% confidence intervals.

**Figure 11:**
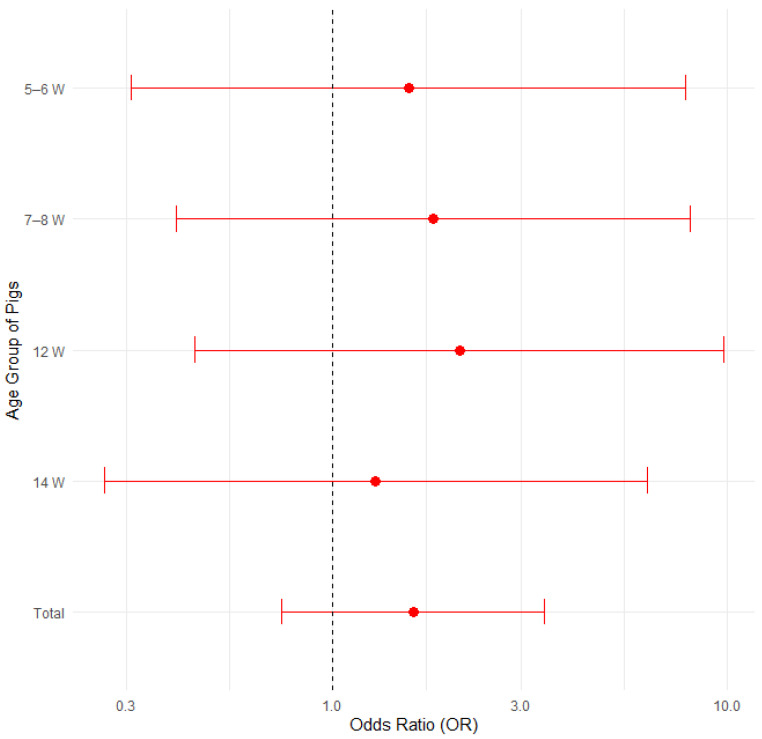
Odds ratios (ORs) with 95% confidence intervals for edema disease presence in farms without past outbreaks vs. farms with past outbreaks.

The presence of edema disease was compared among 22 farms with a history of edema disease and 14 farms with no history of edema disease. Figure 10 illustrates the proportions of positive cases by past outbreaks and pig age group, with 95% confidence intervals in a bar plot, while Figure 11 presents the corresponding odds ratios with their 95% confidence intervals.

There was no statistically significant difference between these two groups when statistically analyzed using Fisher’s exact test for count data, the test of equal or given proportions and the Mann–Whitney test, as shown in the Table 7.

A logistic regression analysis confirmed that neither historical outbreak status nor pig age significantly influenced the likelihood of developing edema disease (all *p* > 0.05, with confidence intervals overlapping 1.0). The results are presented in Table S5 and Figure S5 in the Supplementary Materials.

#### 3.3.6. Prevalence of Edema Disease Based on the Age of Sampled Pigs

**Figure 12:**
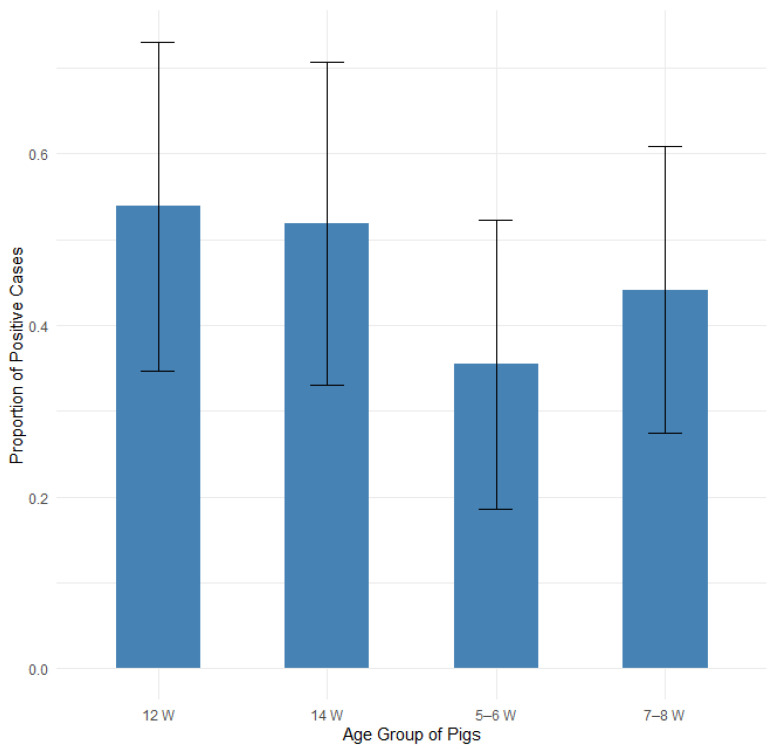
Proportion of positive farms by pig age group with 95% confidence intervals.

**Figure 13:**
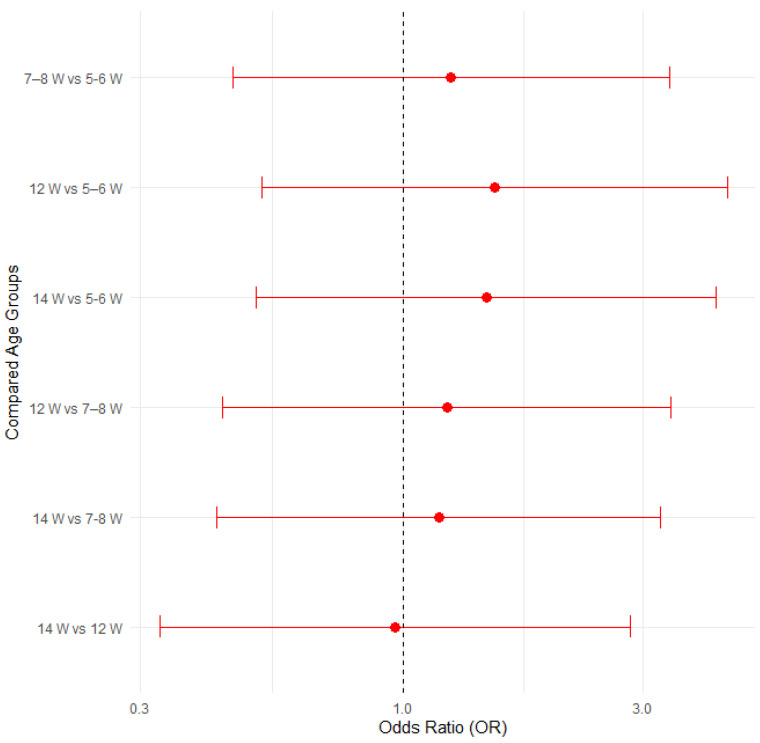
Odds ratios (ORs) with 95% confidence intervals for edema disease presence among different pig age groups.

The proportion of edema disease was compared among groups of 31 pigs (11 positive) aged 5–6 weeks, 34 pigs (15 positive) aged 7–8 weeks, 26 pigs (14 positive) aged 12 weeks and 27 pigs (14 positive) aged 14 weeks. Figure 12 illustrates the proportions of positive cases by pig age group, with 95% confidence intervals in a bar plot, while Figure 13 presents the corresponding odds ratios with their 95% confidence intervals.

None of compared age groups showed a statistically significant difference when analyzed with Fisher’s exact test for count data, the test of equal or given proportions or the Mann–Whitney test (Table 8). 

A logistic regression analysis confirmed that pig age does not significantly influence the likelihood of developing edema disease (all *p* > 0.05, with confidence intervals overlapping 1.0). The results are presented in Table S6 and Figure S6 in the Supplementary Materials.

## 4. Discussion

The aim of this study was to determine the proportion of Slovenian pig farms affected by porcine edema disease and the correlation with the type of pig management and the biosecurity measures applied by the farmers. A total of 37 commercial pig farms were included in this study, where four different age groups of growers and fatteners were non-invasively sampled for oral fluid using Verocheck^®^ diagnostic kits.

The proportion of farms in Slovenia affected by edema disease caused by all strains of *E. coli* with the ability to produce the Stx2e toxin was found to be 64.9%. That is slightly higher that the information from other European countries, such as Germany, where a 53.5% prevalence of the STEC pathotype-producing Stx2e was reported [5], or the USA, where a 65.3% prevalence of the STEC pathotype was reported [20]. An absolutely reliable comparison between the percentages is not possible, as in our study, a pig pen was defined as an epidemiological unit, whereas in the previously mentioned studies, the prevalence was also from individual pig samples. However, one reason for the slightly higher proportion of affected farms in Slovenia and the higher prevalence of edema disease in the USA compared to Germany is that the former two studies used a wider age range of sampled pigs, which further increases the probability of finding and identifying the Stx2e toxin in the pig population. In order to properly compare the results, an individual sampling method for Stx2e should be used in future studies of edema disease in Slovenia.

Non-invasive oral fluid sampling was originally chosen to avoid compromising animal welfare. The simplicity of sampling and inoculating the FTA cards with oral fluid before shipping the samples to Spain was a major advantage over other available non-invasive methods. The one hour required for the pigs to interact with the ropes was used by the sampler to interview the farmers about rearing techniques, biosecurity measures and the general health situation on their farms, while providing the pigs with an enriched environment. Defining a pig pen as an epidemiological unit eliminated the need to handle individual pigs, thereby reducing the stress level during sampling. No problems with ear tags or teeth getting caught in the ropes [5] were observed during our research. The biggest challenge for us was sampling the 5–6-week-old pigs. Compared to the older categories of pigs, they showed less interest in interacting with the ropes and needed more time to make contact with the ropes. Even after prolonged sampling, there was often too little oral fluid to extract from the ropes. This behavioral difference in the youngest pigs, which could not always be overcome by extending the sampling time, could be avoided by introducing another non-invasive sampling method such as fecal sampling or boot swabs, where the sample can be taken regardless of the pigs’ interest in the sampling object. Another sample was lost when the pigs in one of the sampled pens pulled the rope to the ground, where it became contaminated.

Only commercial pig farms with at least 20 breeding sows were used for this study. The majority of the pig herd in Slovenia is distributed among family farms with fewer than 10 pigs of different age groups. Commercial pig farms that meet the criteria of having at least 20 breeding sows are few; therefore, we are aware that the accuracy of our results is limited by the number of samples from farms and pens. The high dispersion of the relatively small pig population in Slovenia might affect the applicability of the prevalence estimates due to unintentional selection bias. To obtain a larger sample, another sampling method such as individual fecal samples or boot swabs, which can be performed independently of the number of pigs per pen, might be applicable.

In Slovenia, edema disease is mainly subclinical, leading farmers to believe that the pathogen is not present on their farms. Pigs that were not vaccinated against edema disease had a 5-fold higher risk of death and a 12-fold higher risk of death in the initial period compared to pigs that were vaccinated with commercially available vaccines against edema disease [27]. Piglets vaccinated with genetically disarmed toxoid Stx2e produced protective antibodies without adverse effects on growth. Immunization of sows with the same toxoid Stx2e protected piglets via colostrum until one month after weaning [28,29]. However, general health data collected from surveys showed that 25 out of 33 farmers did not vaccinate against the EDEC pathotype, resulting in low collective immunity against edema disease on Slovenian pig farms and, thus, a decrease in feed conversion and a higher prevalence of secondary infections [30,31]. There were farmers who had never heard of edema disease and only recognized it from the symptoms we described. This indicates that educational courses on infectious diseases should be organized to improve farmers’ knowledge of the pathogenesis and local prevalence of important pig diseases. In addition, 8 out of 33 interviewed farmers do not vaccinate their pigs against any disease. Together with a low awareness of the need for external and internal biosecurity measures, this leads to an increased possibility of any infection, including edema disease.

Other commonly used strategies for the prevention and control of swine diseases associated with pathogenic *E. coli* strains aim to reduce the number of pathogenic *E. coli* in the environment by implementing appropriate hygiene measures combined with internal and external biosecurity [31]. However, the majority of Slovenian pig farmers purchase clinically healthy pregnant gilts of unknown health status. Out of 33 surveyed farmers, 9 farmers did not have a quarantine for new pigs, 16 farmers did not clean or disinfect the sows before moving them to the nursery and 3 farmers did not clean the fattening pens before moving the pigs there from the nursery. A total of 23 farmers never cleaned the water distribution system, where *E. coli* strains often form highly resistant biofilms [3,32]. This may provide a constant source of infection. Repeated or frequent use of the same type of disinfectant does not increase bacterial resistance to antibiotics and disinfectants [33], so its use is one of the biosecurity measures that play a crucial role in controlling bacterial contamination. Products based on glutaraldehyde/quaternary ammonium compounds and chlorocresol are the most effective against bacteria in microtiter plates [34]. The majority of Slovenian farmers interviewed for this study use Ekocid, which acts as a strong oxidant, while some use Virocid, Agacid, Cid Lines disinfectants, Biomin or a combination of glutaraldehyde and active chloride foam to disinfect pens and sows. 

One of the important triggers of edema disease is the type of feed used [6,12]. It was found that only 4 out of 14 farmers prepared their own feed and knew its ingredients. Feed prepared without any nutritional evaluation may be too high in protein and carbohydrates, leading to increased bacterial colonization of the small intestine. This allows pathogenic *E. coli* serotypes to colonize the villi and produce toxins that are later absorbed through the damaged mucosa [3,6,12]. A total of 7 out of 14 farmers who prepared their own feed did not weigh their pigs. This prevents adequate monitoring of the pigs’ growth level, which could be an early indicator of subclinical edema disease in the case of an unexplained decrease. 

Another factor contributing significantly to an increase in the prevalence of edema disease is the ad libitum feeding method [3]. Especially in summer, the exposure of remaining feed to high ambient temperatures results in poorer hygiene, allowing bacteria to multiply. A total of 27 out of 33 farmers fed their pigs ad libitum, which can lead to the proliferation of pathogenic bacteria despite adequate nutritional compounds in the feed. Dietary preventive measures against edema disease include restrictive feeding practices and the addition of 15–20% fiber to the diet [3], which is problematic because it slows growth and provides too little antigenic substrate for an efficient intestinal immune response against other pathogens [6]. Only 2 out of 33 questioned farmers added etheric oils (oregano, peppermint and fennel), 5 out of 33 added probiotics and 6 out of 33 added organic acids with known beneficial compounds to the diet [3,35]. 

The lack of hygiene in the pens and in the water distribution system, together with unknown feed contents and low collective immunity, probably increase the probability of infection with pathogenic *E. coli* strains. However, contact with a contaminated external environment does not seem to be a very likely factor that would increase the probability of infection if it were the only factor. Although this research did not show a statistically significant association between farm size, previous outbreaks of edema disease or the presence of quarantine and the proportion of edema disease, it cannot be excluded that these factors, when combined, may influence the prevalence. Further research is needed to evaluate this possibility. Other unmeasured factors such as genetic susceptibility or variant *E. coli* strains may have influenced the results. However, pigs without the receptors for the F18 fimbrial adhesive have not yet been massively introduced worldwide [6,13,14], including in Slovenia. The most commonly used breeds in Slovenia are Large White, Landrace, Duroc and their crossbreeds, and they are not yet deliberately selected for immunity to edema disease. Due to the close geographical proximity of the farms participating in this study, it is likely that all analyzed pigs originate from a similar genetic pool. The EDEC pathotype associated with edema disease may not be the only causative pathotype; interbacterial exchange of genetic material that would allow other strains to encode and produce Stx2e and cause (sub)clinical edema disease [6,7,8] cannot be excluded without further research. Verdonck et al. [36] reported a significantly higher number of seropositive open pig farms compared to closed farms, showing that the importance of herd management and external biosecurity may vary between regions. More closed breeding farms need to be identified and included in future research. Transmission of pathogenic *E. coli* strains responsible for edema disease between wild boars and domestic pigs in outdoor pens, as reported in France by Jori et al. [37], is unlikely in Slovenia according to the results of this study. One of the reasons for this could be the increased awareness of external biosecurity due to African swine fever prevention measures. Biological reasons for a positive association between herd size and swine diseases include a greater risk of pathogen introduction from the external environment or transmission within and between large herds [38]. In contrast to other infectious diseases, such as swine influenza, where there is a proven positive association between farm size and disease prevalence [39], we found no significant association between the proportion of edema disease and farm size, which may mean that the effect of herd size alone is not significant. 

The high proportion of subclinical edema disease is probably closely related to the low frequency of preventive and diagnostic sampling. The lack of interest in the diagnosis of infectious swine diseases is most likely related to the high prices and long waiting times for results associated with traditional microbiological methods, as well as a lack of knowledge of the pathogenesis of infectious diseases among farmers. However, the development of non-invasive sampling methods, such as the oral fluid collection method used in this study, may eventually encourage farmers to test their pigs more frequently for infectious diseases, thereby detecting, treating and preventing diseases earlier. As there are no official databases on the prevalence of edema disease in Slovenia, information on past outbreaks of edema disease was only collected through the questionnaire. If farmers and veterinarians were more involved in the sampling and diagnosis of infectious diseases, they would have access to more relevant data on disease outbreaks, which could further aid future research. Due to the uneven distribution of the pig population, the use of a different type of non-invasive individual sampling, as opposed to simply extending the duration of the oral fluid sampling method in the pen, may be more efficient for the sampling of smaller populations, including those of non-commercial farms. By better understanding the incidence of edema disease caused by Stx2e-producing *E. coli* virotypes in Slovenia, we hope to help encourage farmers to undertake biosecurity and preventive measures against edema disease.

Contrary to theoretical information on the distribution of edema disease [9], we found no statistically significant difference in prevalence when comparing different age groups of pigs. Pathogenic *E. coli* strains with the ability to produce the Stx2e toxin were found in all age groups of pigs, with none of them showing a statistically significantly higher prevalence when compared to other age groups. This may be due to a general lack of hygiene, which allows the formation of resistant *E. coli* biofilms in water distribution systems and pig pens. Although this does not cause the clinical course of edema disease, there are still significant losses associated with slower growth and an increase in other infectious diseases due to a weakened immune system. However, these consequences of subclinical infections are unknown to farmers who do not measure the body weight and feed conversion of their animals. In the future, further studies are needed to compare the prevalence of edema disease caused by the EDEC pathotype with other *E. coli* pathotypes that also have the ability to produce the Stx2e toxin, possibly by using different non-invasive sampling methods and including smaller epidemiological units within the Slovenian pig population. 

## 5. Conclusions

In Slovenia, the current proportion of edema disease caused by all *E. coli* serotypes that have the ability to produce the Stx2e toxin is 64.9%. The proportion of edema disease was statistically significantly higher on farms where the farmer prepares the feed himself and where the pigs do not have access to outdoor runs, while there was no statistically significant difference in relation to farm size, previous outbreaks of edema disease or the presence of the quarantine for newly purchased pigs. Contrary to the current data, there was no statistically significant correlation between the age of growers and fatteners and the proportion of edema disease on Slovenian farms, indicating that the distribution of the disease is not higher in the days immediately after weaning of piglets or that the age of the pigs may not be one of the most important factors influencing the proportion of the disease. Non-invasive sampling of oral fluid to assess the presence of edema disease has been shown to be generally effective, opening up the possibility of encouraging farmers to sample pigs more frequently for infectious diseases and to record production and environmental parameters more carefully in the future. Further studies are needed to determine the relationship between edema disease cases caused by the EDEC serotype and edema disease caused by other pathogenic serotypes that also have the ability to produce the Stx2e toxin. However, the limited sample size may have affected the ability to detect true associations, and further research with larger populations is recommended.

## Figures and Tables

**Table 1:** Information on the farms and their technology, collected through surveys.

Farm	Region	Type	Size	Quarantine	Feed	ED
1	Š	O	700	−	N	+
2	Š	I	1200–1400	−	N	+
3	P	I	1100	+	C	+
4	P	I	780	−	C	+
5	Š	O	691	+	C	+
6	Š	I	242	+	C	+
7	Š	I	390	−	N	+
8	P	O	500	+	C	+
9	P	I	340	+	C	+
10	Š	O	300	+	N	+
11	Š	O	835	−	C	+
12	Š	O	352	+	C	+
13	P	I	315	+	N	+
14	Š	O	<500	+	C	+
15	P	I	385	+	C	−
16	P	I	740	−	C	−
17	P	O	703	+	C	−
18	P	O	980	+	C	−
19	Š	I	720	−	C	−
20	Š	O	2500	+	C	−
21	Š	I	1384	+	N	−
22	P	/	/	/	/	/
23	Š	I	>500	+	N	+
24	Š	O	<500	+	N	+
25	D	I	9200	+	C	+
26	Š	I	2258	+	N	+
27	Š	O	3200	+	C	−
28	D	I	17,500	+	C	−
29	Š	I	>500	−	N	−
30	Š	O	121	−	N	−
31	Š	I	598	−	N	+
32	Š	I	550	+	C	+
33	Š	I	700	+	N	−
34	Š	I	300	+	N	+
35	Š	O	220	−	N	−
36	Š	I	188	+	N	−
37	Š	I	450	−	N	−

Legend for Table 1: Farms from three regions were surveyed: Štajerska (Š), Prekmurje (P) and Dolenjska (D). Based on the type of farming, they were divided groups consisting of farms where the pigs had no access to outdoor runs (I) and those where the pigs had access to outdoor runs (O). The farmers were asked about the size of the farm, the criteria being the total number of pigs of all categories on the farm, with (+) indicating the presence of quarantine and confirmed previous outbreaks of edema disease (ED) on the farm and (−) indicating the absence of quarantine and previously confirmed outbreaks of edema disease. The farmers were also asked about the origin of the feed, dividing the farms into groups where the pigs were fed with commercial feed (C) and those where they were fed meals prepared by farmers themselves from home-grown cereals mixed with commercial premixes (N).

**Table 2:** Results of RT-PCR analysis of oral fluid samples from pigs aged 5–6 weeks, 7–8 weeks, 12 weeks and 14 weeks.

Farm	5–6 W	7–8 W	12 W	14 W
1	−	++	−	−
2	+/++	+/+	+/+	−/+
3	0	+++/+++	0	0
4	−	−	0	−
5	−	−	−	0
6	−	−	+	+
7	−	+	++	++
8	0	+++	+	++
9	+++	+++	+	+
10	0	0	0	−
11	−	−	−	−
12	−	−/−	0	0
13	−	++/++	++	0
14	−	−	0	−
15	0	−	0	+
16	−	−	−	+
17	−	−	+	−
18	−	−	−	−
19	−	−	−	−
20	−/−/−/−/−	−/−	−/−/−/−/−/−	0
21	−/−	−/−	−/−	0
22	++	+	++	−
23	++	++	++	++
24	−	−	0	0
25	−/−/−/−/−	0	−/−/−/−/−	+/−/−/−/−
26	++/+++	++	0	0
27	+/−/++/+	+++/+++/+++/++	0	+/−/−/−
28	−/−/−/−/−	−/−/−/−/−	0	−/−/−
29	0	+	+++	0
30	−	−	−	−
31	+	−	+	+
32	+	−	−	−
33	0	+	++	+
34	+++	0	++	++
35	++	++	+	0
36	++	++	0	+
37	−	−	−	−

Legend for Table 2: (−) indicates that the RT-PCR assay did not detect any genetic material of the tested pathogen in the sample, (+) indicates that the RT-PCR assay detected a small amount of genetic material of the tested pathogen, (++) indicates that the RT-PCR assay detected a moderate amount of genetic material of the tested pathogen, (+++) indicates that the RT-PCR assay detected a large amount of genetic material of the tested pathogen and (0) indicates that there was no sample to test for the corresponding age group. Where more than one sample per age group was tested, the individual results of the RT-PCR assay are separated by (/).

**Table 3:** Comparison of the proportion of farms affected by farm size in different age groups of pigs.

Age Group	+ No. L	No. L	+ No. S	No. S	Fisher’s *p*-Value	Proportions *p*-Value	Mann–Whitney *p*-Value
5–6 W	6	17	4	13	1.00	0.85	0.82
7–8 W	8	18	5	13	1.00	0.83	0.76
12 W	6	16	8	11	0.12	0.32	0.08
14 W	7	14	7	12	0.71	0.82	0.70
Total	27	65	24	49	0.45	0.63	0.43

Legend of Table 3: (+ No. L) is the number of samples from large farms that tested positive for edema disease, (No. L) is the total number of samples from large farms, (+ No. S) is the number of samples from small farms that tested positive for edema disease and (No. S) is the total number of samples from small farms. (Fisher *p*-value) stands for the *p*-value of Fisher’s exact test for count data, (Proportions *p*-value) stands for the *p*-value of the test of equal or given proportions and (Mann–Whitney *p*-value) stands for the *p*-value of the Mann–Whitney test.

**Table 4:** Effect of husbandry and farm size on the occurrence of disease.

Age Group	+ No. I	No. I	+ No. O	No. O	Fisher’s *p*-Value	Proportions *p*-Value	Mann–Whitney *p*-Value
5–6 W	9	20	1	10	0.10	0.15	0.06
7–8 W	11	21	2	10	0.13	0.25	0.10
12 W	12	20	2	7	0.21	0.39	0.17
14 W	14	19	0	7	0.001	0.03	0.001
Total	46	80	5	34	0.00003	0.005	0.00003

Legend for Table 4: (+ No. I) is the number of samples from farms where pigs only had access to indoor pens that tested positive for edema disease, (No. I) is the number of all samples from farms where pigs only had access to indoor pens, (+ No. O) is the number of samples from farms where pigs had access to indoor and outdoor pens that tested positive for edema disease and (No. O) is the number of all samples from farms where pigs had access to both indoor and outdoor pens. (Fisher *p*-value) stands for the *p*-value of Fisher’s exact test for count data, (Proportions *p*-value) stands for the *p*-value for the test of equal or given proportions, and (Mann–Whitney *p*-value) stands for the *p*-value of the Mann–Whitney test.

**Table 5:** Effect of the type of feed in different age groups of pigs on disease presence.

Age Group	+ No. C	No. C	+ No. O	No. O	Fisher’s *p*-Value	Proportions *p*-Value	Mann–Whitney *p*-Value
5–6 W	3	16	7	14	0.12	0.20	0.08
7–8 W	4	16	9	15	0.07	0.21	0.06
12 W	3	13	10	14	0.02	0.13	0.01
14 W	7	15	8	11	0.25	0.50	0.20
Total	17	60	34	54	0.0003	0.02	0.0002

Legend for Table 5: (+ No. C) is the number of samples from farms where only commercial feed was used that tested positive for edema disease, (No. C) is the total number of samples from farms where only commercial feed was used, (+ No. O) is the number of samples from farms where farmers prepared feed on their own that tested positive for edema disease and (No. O) is the total number of samples from farms where farmers prepared feed on their own. (Fisher *p*-value) stands for the *p*-value of Fisher’s exact test for count data, (Proportions *p*-value) stands for the *p*-value of the test of equal or given proportions, and (Mann–Whitney *p*-value) stands for the *p*-value of the Mann–Whitney test.

**Table 6:** Effect of quarantine in different age groups of pigs on edema disease proportion.

Age Group	+ No. Q	No. Q	+ No. W	No. W	Fisher’s *p*-Value	Proportions *p*-Value	Mann–Whitney *p*-Value
5–6 W	6	17	4	12	1.00	0.94	0.94
7–8 W	7	17	6	13	1.00	0.86	0.81
12 W	7	15	6	11	1.00	0.82	0.72
14 W	10	14	4	11	0.12	0.34	0.09
Total	30	63	20	47	0.70	0.75	0.60

Legend for Table 6: (+ No. Q) is the number of samples from farms where biosecurity included quarantine that tested positive for edema disease, (No. Q) is the total number of samples from farms where biosecurity included quarantine, (+ No. W) is the number of samples from farms where quarantine was not included in biosecurity that tested positive for edema disease and (No. W) is the total number of samples from farms where quarantine was not included in biosecurity measures. (Fisher *p*-value) stands for the *p*-value of Fisher’s exact test for count data, (Proportions *p*-value) stands for the *p*-value of the test of equal or given proportions and (Mann–Whitney *p*-value) stands for the *p*-value of the Mann–Whitney test.

**Table 7:** Effect of presence of past outbreaks of edema disease in different age groups of pigs on disease proportion.

Age Group	+ No. E	No. E	+ No. N	No. N	Fisher’s *p*-Value	Proportions *p*-Value	Mann–Whitney *p*-Value
5–6 W	7	19	3	11	0.70	0.70	0.62
7–8 W	9	19	4	12	0.48	0.62	0.46
12 W	9	15	5	12	0.45	0.59	0.37
14 W	9	16	5	10	1.00	0.86	0.78
Total	34	69	17	45	0.70	0.45	0.23

Legend for Table 7: (+ No. E) is the number of samples from farms that had encountered edema disease outbreaks in the past and tested positive for edema disease, (No. E) is the total number of samples from farms that had encountered edema disease outbreaks in the past, (+ No. N) is the number of samples from farms that had never encountered outbreaks of edema disease in the past and tested positive for edema disease, (No. N) is the total number of samples from farms that had never encountered edema disease outbreaks before. (Fisher *p*-value) stands for the *p*-value of Fisher’s exact test for count data, (Proportions *p*-value) stands for the *p*-value of the test of equal or given proportions and (Mann–Whitney *p*-value) stands for the *p*-value of the Mann–Whitney test.

**Table 8:** Effect of different age groups of pigs on disease proportion.

Compared Age Groups	Fisher’s *p*-Value	Proportions *p*-Value	Mann–Whitney *p*-Value
7–8 W vs. 5-6 W	0.61	0.66	0.51
12 W vs. 5–6 W	0.20	0.38	0.17
14 W vs. 5-6 W	0.29	0.43	0.22
12 W vs. 7–8 W	0.61	0.65	0.46
14 W vs. 7-8 W	0.61	0.71	0.54
14 W vs. 12 W	1.00	0.94	0.91

## Data Availability

The original contributions presented in the study are included in the article; further inquiries can be directed to the corresponding author.

## Supplementary Materials

The following supporting information can be downloaded at: https://www.mdpi.com/article/10.3390/pathogens14030261/s1, Table S1. Logistic regression results: effect of the association between farm size and age groups of pigs on the occurrence of diseases; Figure S1: Odds ratios (log scale) with 95% confidence intervals from logistic regression assessing the effect of farm size and pig age groups on edema disease proportion; Table S2. Logistic regression results: effect of type of husbandry on edema disease proportion; Figure S2: Odds ratios (log scale) with 95% confidence intervals from logistic regression assessing the effect of type of husbandry on edema disease proportion; Table S3. Logistic regression results: effect of feed type on edema disease proportion; Figure S3: Odds ratios (log scale) with 95% confidence intervals from logistic regression assessing the effect of feed type on edema disease proportion; Table S4. Logistic regression results: effect of quarantine on edema disease proportion; Figure S4: Odds ratios (log scale) with 95% confidence intervals from logistic regression assessing the effect of quarantine on edema disease proportion; Table S5: Logistic regression results: effect of past outbreaks on edema disease proportion; Figure S5: Odds ratios (log scale) with 95% confidence intervals from logistic regression assessing the effect of past outbreaks on edema disease proportion; Table S6. Logistic regression results: effect of pig age on edema disease proportion; Figure S6: Odds ratios (log scale) with 95% confidence intervals from logistic regression assessing the effect of pig age on edema disease proportion.
