# Supplementary material for: Cross-Sectional Study: Assessing the Presence of Stx2e-Producing E. coli Virotypes in Samples of Oral Fluid of Growers and Fatteners
Source: Pathogens. 2025 Mar 6;14(3):261. doi: 10.3390/pathogens14030261 (PMC11944655; doi:10.3390/pathogens14030261)
Supplement: Supplementary file 1 [file pathogens-14-00261-s001.zip › pathogens-3456591-supplementary.pdf]

Table S1. Logistic regression results: association between farm size and age group of pigs on the occurrence of diseases.

Coefficient	Estimate	P value	Odds Ratio	CI Lower	CI Upper
(Intercept)	-0.83	0.052	0.44	0.18	0.98
small vs large	0.31	0.43	1.36	0.64	2.91
7-8W vs 5-6W	0.37	0.48	1.45	0.51	4.21
12W vs 5-6W	0.78	0.15	2.18	0.75	6.55
14W vs 5-6W	0.84	0.13	2.32	0.80	7.06

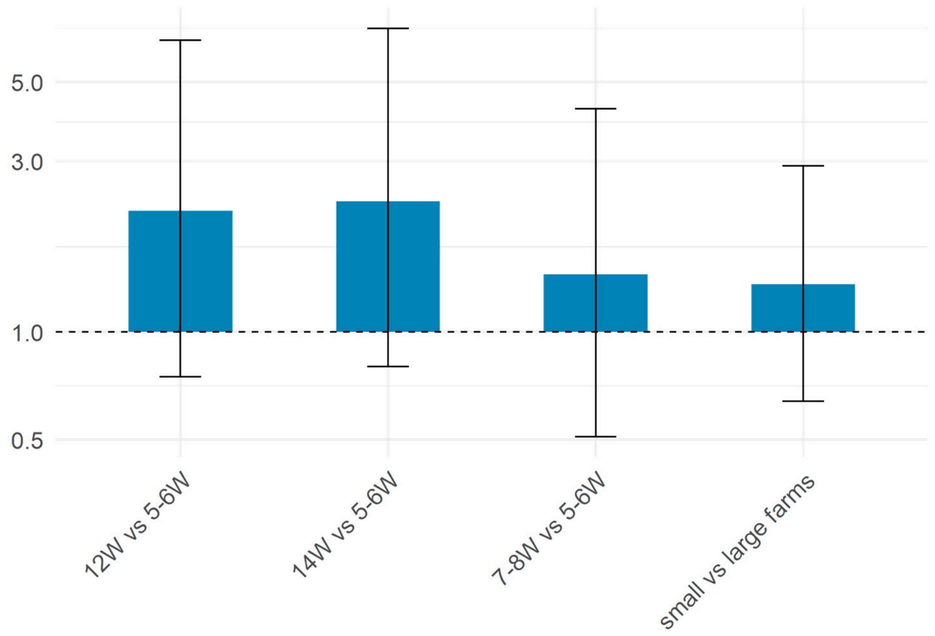

Figure S1: Odds Ratios (log scale) with 95% Confidence Intervals from logistic regression assessing the effect of farm size and pig age group on edema disease proportion.

Table S2. Logistic regression results: effect of type of husbandry on edema disease proportion.

Coefficient	Estimate	P value	Odds Ratio	CI Lower	CI Upper
(Intercept)	-0.19	0.65	0.83	0.35	1.88
outdoor vs indoor	-2.06	0.0001	0.13	0.04	0.34
7-8W vs 5-6W	0.41	0.48	1.50	0.49	4.72
12W vs 5-6W	0.75	0.20	2.12	0.67	6.92
14W vs 5-6W	0.87	0.15	2.38	0.75	7.96

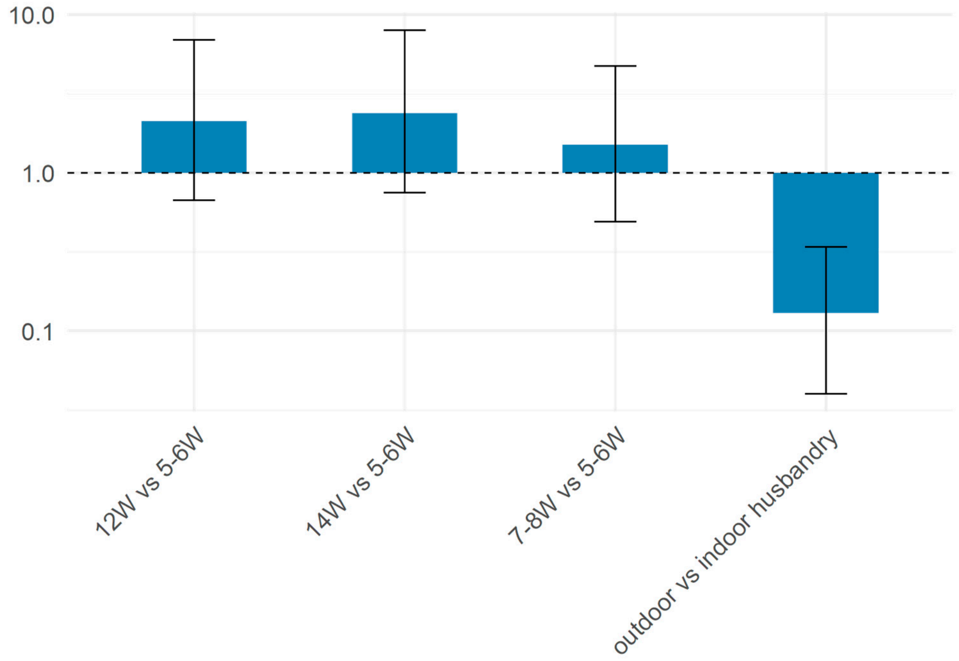

Figure S2: Odds Ratios (log scale) with 95% Confidence Intervals from logistic regression assessing the effect of type of husbandry on edema disease proportion.

Table S3. Logistic regression results: effect of feed type on edema disease proportion.

Coefficient	Estimate	P value	Odds Ratio	CI Lower	CI Upper
(Intercept)	-1.52	0.002	0.22	0.08	0.54
own vs commercial	1.55	0.0002	4.72	2.12	11.02
7-8W vs 5-6W	0.39	0.49	1.48	0.49	4.61
12W vs 5-6W	0.62	0.29	1.87	0.60	6.02
14W vs 5-6W	1.23	0.04	3.42	1.08	11.58

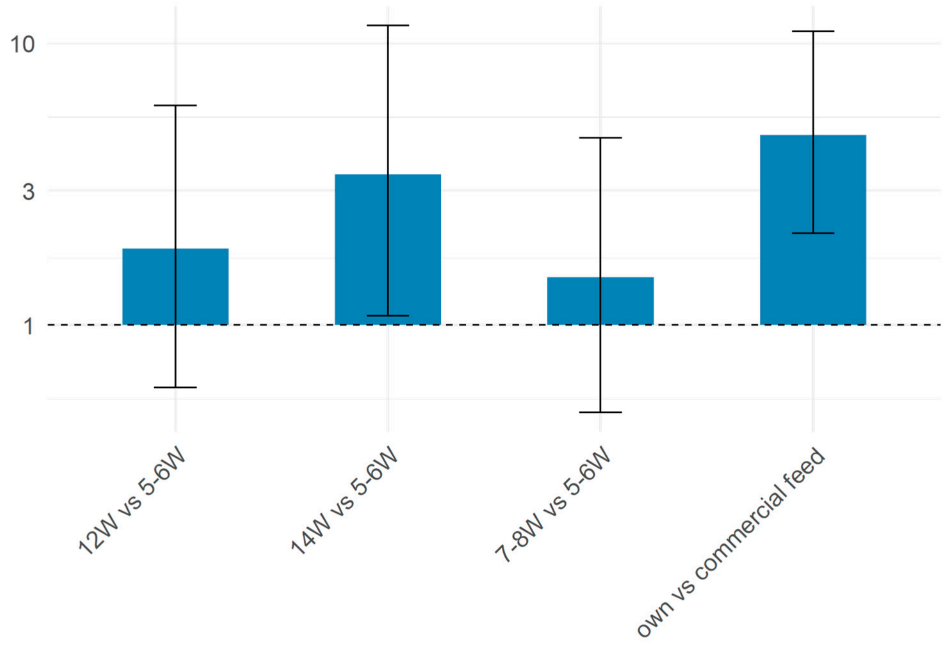

Figure S3: Odds Ratios (log scale) with 95% Confidence Intervals from logistic regression assessing the effect of feed type on edema disease proportion.

Table S4. Logistic regression results: effect of quarantine on edema disease proportion.

Coefficient	Estimate	P value	Odds Ratio	CI Lower	CI Upper
(Intercept)	-0.77	0.09	0.46	0.18	1.11
with vs without quarantine	0.22	0.57	1.25	0.58	2.72
7-8W vs 5-6W	0.38	0.48	1.46	0.51	4.27
12W vs 5-6W	0.65	0.24	1.91	0.65	5.78
14W vs 5-6W	0.89	0.11	2.44	0.82	7.56

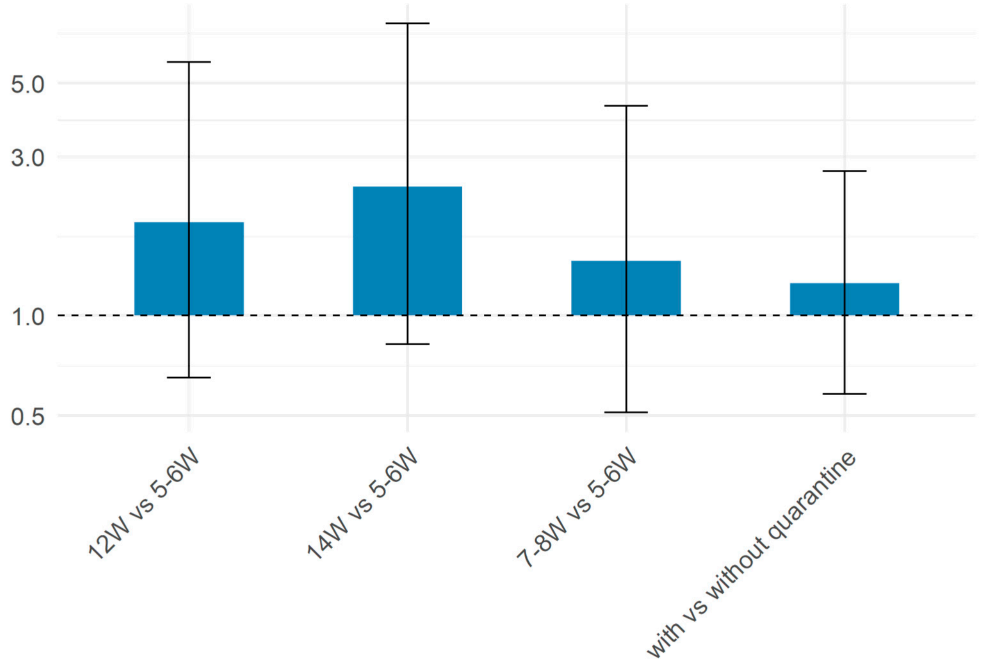

Figure S4: Odds Ratios (log scale) with 95% Confidence Intervals from logistic regression assessing the effect of quarantine on edema disease proportion.

Table S5. Logistic regression results: effect of past outbreaks on edema disease proportion.

Coefficient	Estimate	P value	Odds Ratio	CI Lower	CI Upper
(Intercept)	-0.52	0.21	0.60	0.26	1.31
without vs with past outbreaks	-0.51	0.20	0.60	0.27	1.30
7-8W vs 5-6W	0.38	0.47	1.47	0.52	4.27
12W vs 5-6W	0.82	0.14	2.27	0.78	6.88
14W vs 5-6W	0.87	0.12	2.38	0.81	7.30

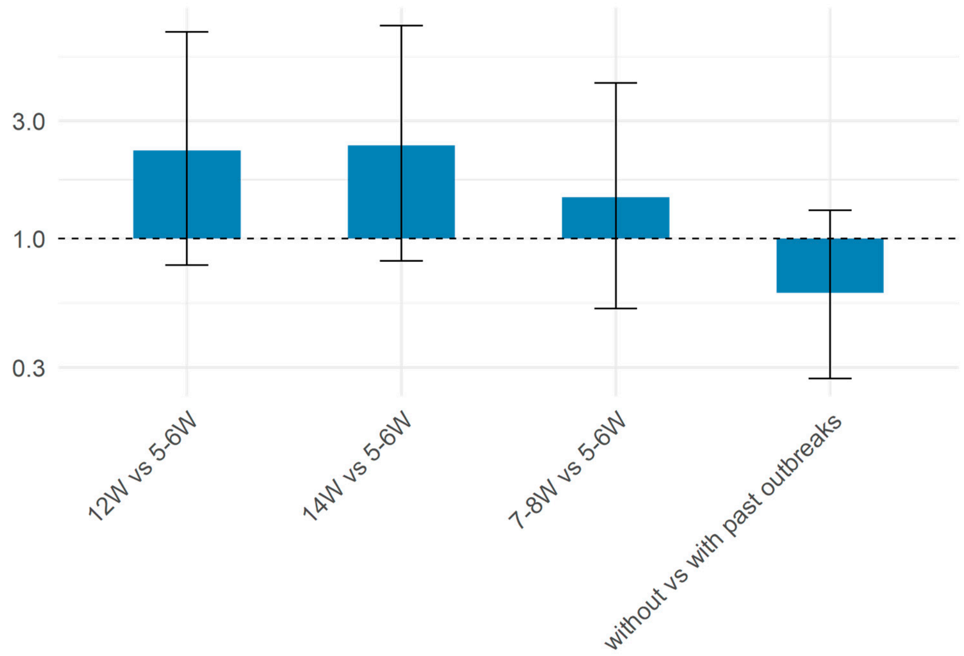

Figure S5: Odds Ratios (log scale) with 95% Confidence Intervals from logistic regression assessing the effect of past outbreaks on edema disease proportion.

Table S6. Logistic regression results: effect of pig age on edema disease proportion.

Coefficient	Estimate	P value	Odds Ratio	CI Lower	CI Upper
(Intercept)	-0.60	0.11	0.55	0.25	1.13
7-8W vs 5-6W	0.35	0.50	1.41	0.51	3.96
12W vs 5-6W	0.74	0.16	2.10	0.75	6.10
14W vs 5-6W	0.67	0.21	1.96	0.69	5.74
total vs 5-6W	0.43	0.31	1.53	0.69	3.58

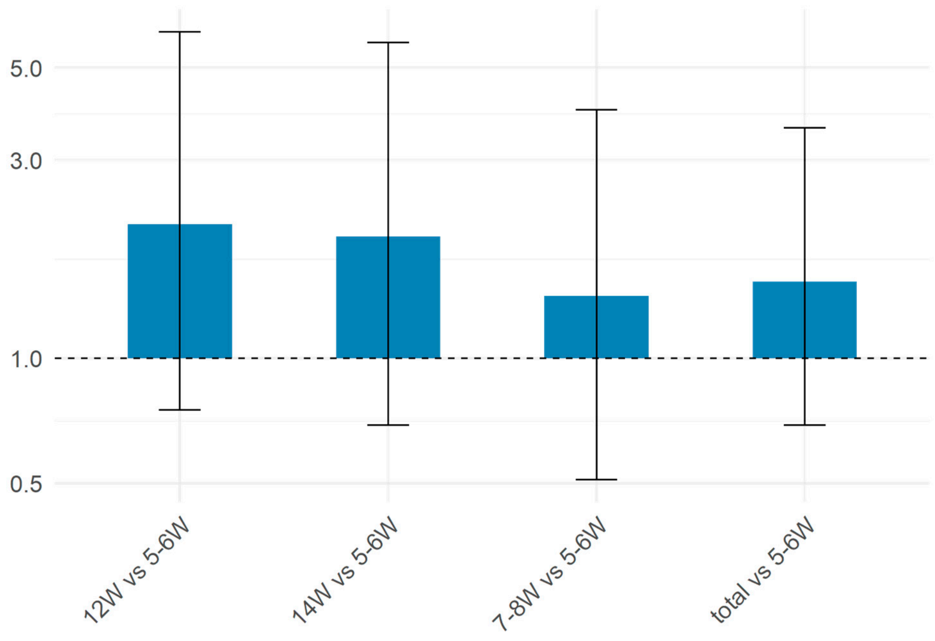

Figure S6: Odds Ratios (log scale) with 95% Confidence Intervals from logistic regression assessing the effect of pig age on edema disease proportion.
